# Dietary probiotic supplementation with *Lactococcus lactis* and *Bacillus velezensis* enhances growth performance, meat quality, blood profiles, and cecal and feed microbiota in growing rabbits

**DOI:** 10.1080/10495398.2025.2577947

**Published:** 2025-10-31

**Authors:** Nada Elbaz, Elwy A. Ashour, Samar S. Bassiony, Ahmed I. Elsherbeni, Samir A. Mahgoub, Ali O. Osman, Islam Sabike, Naif A. Al-Gabri, Mahmoud Moustafa, Mohammed Al-Shehri, Mohamed E. Abd El-Hack

**Affiliations:** ^a^ Poultry Department, Faculty of Agriculture, Zagazig University, Zagazig, Egypt; ^b^ Animal Production Systems Research Department, Animal Production Research Institute, Agricultural Research Center, Dokki, Giza, Egypt; ^c^ Department of Microbiology, Faculty of Agriculture, Zagazig University, Zagazig, Egypt; ^d^ Biochemistry Department, Faculty of Agriculture, Zagazig University, Zagazig, Egypt; ^e^ Department of Food Hygiene and Control, Faculty of Veterinary Medicine, Benha University; ^f^ Department of Pathology, Salam Central Laboratory, Salam Veterinary Group, Buraydah 51911, Saudi Arabia; ^g^ Department of Biology, College of Science, King Khalid University, Abha, Kingdom of Saudi Arabia; ^h^ Department of Industrial Pharmacy, College of Pharmaceutical Sciences and Drug Manufacturing, Misr University for Science and Technology (MUST), Giza, Egypt

**Keywords:** Probiotic, New Zealand White rabbits, growth performance, meat quality, microbial load

## Abstract

This study explored the effects of dietary supplementation with a probiotic blend of *Lactococcus lactis* subsp. *lactis* and *Bacillus velezensis* on the performance and health of growing rabbits. Ninety-six rabbits were allocated into three groups and fed for eight weeks on diets containing either no probiotic (control), a low-dose probiotic (1 ml/kg of each probiotic strain), or a high-dose probiotic (2 ml/kg of each probiotic strain). Probiotic inclusion, particularly at the higher level, significantly enhanced final body weight, feed conversion ratio, and overall performance. Improvements were also noted in carcass characteristics, with greater yields in both forequarters and hindquarters. Blood biochemical analysis revealed elevated antioxidant enzyme activity and immunoglobulin levels, alongside reductions in lipid fractions and better liver and kidney function indicators. Meat quality also benefited, as shown by lower pH, reduced water holding capacity and cooking loss, and more favorable color metrics. Microbial analysis of cecal and feed samples indicated a notable increase in beneficial bacteria and a decline in pathogenic species in the probiotic-treated groups. These findings suggest that combined probiotic supplementation is a promising strategy for enhancing growth performance, meat quality, and gut health in rabbit production systems to produce a good final meat product.

## HIGHLIGHTS

1. Probiotics improved growth performance and feed efficiency in NZW rabbits.
2. Enhanced carcass yields and meat quality were observed.
3. Antioxidant status and lipid profile were significantly improved.
4. Beneficial gut microbes increased while pathogens decreased.
5. A higher probiotic dose showed the most pronounced effects.

## Introduction

In most developing nations, feeding problems are still a nightmare, especially since animal protein production is declining and making malnutrition worse.^2^ Achieving food security worldwide and satisfying the rising demand for meat may be achieved by improving rabbit production. Humans can consume high-quality rabbit meat, and they may be a significant way to address some of the problems linked with the meat shortage.^3^ To meet the increasing demand for meat and ensure global food security, rabbit production has become crucial.^4^ Rabbits were considered a good source of animal-derived meat and proteins; they can help minimize the gap of animal protein reduction, due to their excellent fertility and rapid growth rates.^5^ Low-calorie content, enough animal protein, and polyunsaturated fatty acids are excellent characteristics of rabbit meat.^6^ To enhance growth performance and feed efficiency, natural feed additives were employed in poultry and ruminant diets.^7^ Conventional antibiotics were frequently employed in livestock and poultry farms to avoid or treat infections caused by various bacterial species in the past. Nevertheless, the excessive use and abuse of these medications have had major adverse effects, such as the emergence of antibiotic-resistant bacterial strains, which pose a significant concern to human health at the moment (Abreu et al. 2023).^8^^,^^9^ The price and quality of diet components, which account for 60–70% of overall operating costs, are a major worldwide issue for animal Production, thus employing natural resources in poultry diets has gained popularity recently.^10^^,^^11^ Discovering efficient antibiotic alternatives in rabbit nutrition was urgently needed. Environmentally conscious feed additives are becoming increasingly utilized to boost animal growth and health.^12^^,^^13^

Probiotics, or beneficial bacterial species, are becoming increasingly popular as a safe alternative to antibiotics. The word ‘probiotic’ was coined in 1965 to refer to a chemical generated by one organism that promotes the growth of other bacteria.^14^ Probiotics mostly consist of lactic acid bacteria like *Lactococcus*, *Enterococcus*, *Pediococcus*, *Streptococcus*, and others.^15^ Providing diets with probiotics and prebiotics has been shown to boost feed utilization, intestinal strength, and rates of growth performance.^16–18^ Their ability to support the immune system function, optimize consumption rates of diet, prevent potentially disease-causing infections, and keep gut microbiota healthy further highlights their probable to maximize health and production of animals.^19^

Most of the probiotics’ benefits can be summarized as follows: (I) increasing antioxidative capacity; (II) improving digestion as well as absorption of dietary nutrients; (III) strengthening immunity; (IV) maintaining gut health and functionality; and (V) establishing an advantageous gut microbiota balance.^20^^,^^21^ Furthermore, probiotics are a safe antibiotic alternative, which was confirmed by El-Sawy et al.^22^ who found that probiotics inclusion in rabbit drinking water boosted growth performance and FCR in rabbits without negatively impacting carcass characteristics. Previous studies noted that probiotics addition might lessen the rates of death in rabbits, particularly during the dangerous period of weaning.^23^

As well, probiotics supplementation for rabbits enhanced most of their carcass traits; besides serum total cholesterol (TC), low-density lipoprotein (LDL), high-density lipoprotein (HDL), and triglycerides (TG) all considerably reduced.^24^ Numerous investigations have shown that probiotic species can control the intestinal microbiota’s variation and activity, which enhances intestinal health as well as epithelial function.^25–27^ Probiotics may interact with the host’s intestinal microbiota, potentially preventing harmful bacteria from developing.^28^ Therefore, assessing the influence of probiotics as feed supplements on rabbits’ growth performance traits, blood indices, body antioxidant status, and equilibrium modification of the cecal microbiota population was the main goal of this study.

## Materials and methods

### Animals and experimental design

This experiment continued for approximately two months at the Department of Poultry, Faculty of Agriculture, Zagazig University, Zagazig, Egypt, during the period from April 2025 to May 2025. The Institutional Animal Care and Use Committee’s rules were followed when conducting the experiments (ZU-IACUC). They were approved by Zagazig University’s Institutional Ethics Committee in Zagazig, Egypt.

In an open-system building, a total number of 96 New Zealand White (NZW) growing rabbits (weighing approximately 844.00 ± 1.85g) at the age of weaning (35 days) were housed collectively (4 rabbits/cage) in one-level wire cages with an interior space of 20 cm^2^ per rabbit, and LED lights were used to extend the photoperiod up to 16 hours during the experiment period. Three experimental groups (32 growing rabbits each group) were randomly assigned to eight replicates of four rabbits each. A completely randomized design was performed, including 3 experimental groups. The 1st group (T0) was growing rabbits that didn’t receive probiotics, the 2nd group (T1) was treated with 1 ml of *Bacillus velezensis +*1 ml of *Lactococcus lactis subsp. Lactis* fresh culture/kg of growing rabbits’ diet, and the 3rd group (T2) was treated with 2 ml of *Bacillus velezensis +* 2 ml of *Lactococcus lactis* subsp*. lactis* fresh culture/kg of rabbits’ diet. The concentration of fresh culture suspension of both bacteria types was 1.3 × 10^8^ and 1.34 × 10^8^ CFU/mL, respectively.

### *Preparation of* Basillus velezensis *and* Lactococcus lactis *probiotics for feeding*

Probiotic bacteria are strains of *Bacillus velezensis* and *Lactococcus lactis* subsp*. lactis* was achieved from the Department of Agricultural Microbiology at the Faculty of Agriculture, Zagazig University, Zagazig, Egypt.

Probiotic bacteria were grown in MRS medium (Man, Rogosa, and Sharpe, Biolife, Italy) containing 10 g/L Peptone, 10 g/L Beef Extract, 4 g/L Yeast Extract, 20 g/L Glucose, 5 g/L Sodium Acetate Trihydrate, 1 g/L Polysorbate 80 (Tween 80), 2 g/L Dipotassium Hydrogen Phosphate, 2 g/L Triammonium Citrate for 36 h at 35 ^0^C. After development, each culture was performed for centrifugation at 12,000 × G to achieve living cells. The cells were washed three times in a row and sent to achieve only live cells each time. Then a suspension of living cells was made in a saline (8.5 g NaCl/l) so that the concentration of cells in this salt solution was 1.3 × 10^8^ and 1.34 × 10^8^ CFU/ml for *Basillus velezensis* and *Lactococcus lactis* probiotics, respectively. This inoculum was used only from living cells of probiotics and supplemented for the feed. Thus, they avoid errors caused by the medium.

### Housing, feeding, and environment

The tested rabbits were kept in 80 × 50 × 40 cm galvanized wire cage batteries with windows providing adequate natural ventilation (which are open-housing systems). Throughout the experimental periods, the ambient temperature ranged between 23 °C and 35 °C, and the relative humidity (RH%) was kept between 51% and 63%. With free access to diets and fresh water. The rabbits were fattened until 77 days (11 weeks) old. Cages were provided with stainless feeders and drinking nipples. Day by day, all rabbits were checked on, found to be healthy, clinically free of parasites both inside and outside, against prevalent diseases, all rabbits were vaccinated, and housed in equal hygienic and management status. The diets were iso-nitrogenous (16.30% Crude Protein) and iso-caloric (2600 kcal Digestible Energy/kg diet), formulated according to NRC,^29^ and according to AOAC.^30^ The chemical composition of the basal diet is displayed in Table 1.

**Table 1.** Ingredients and chemical composition of the basal diet used during the experimental period.

Items	Content (g/kg)
Ingredients	
Barley	320.0
Wheat Bran	210.0
Soy bean Meal (44%)	100.0
Hay	220.0
Berseem Straw	60.0
Decorticated Cottonseed Meal	30.0
Molasse	30.0
Limestone	10.0
Salt	3.40
Minerals and Vitamins Concentrate:*	3.0
DL-Methionine	0.60
Anti-Coccidian and Antibiotic.	13.0
Total	1000
Calculated analysis:**	
Crude Protein	16.30
Crude Fiber	13.20
Ether Extract	2.50
Digestible Energy, K Cal / Kg	2600

* Premix provided per kg of complete diet: vitamin A, 12,000 IU; vitamin D3, 1000 IU; vitamin E acetate, 50 mg; vitamin K3, 2 mg; biotin, 0.1 mg; thiamin, 2 mg; riboflavin, 4 mg; vitamin B6, 2 mg; vitamin B12, 0.1 mg; niacin, 40 mg; pantothenic acid, 12 mg; folic acid, 1 mg; choline chloride, 300 mg; iron, 100 mg; copper, 20 mg; magnesium, 50 mg; cobalt, 2 mg; iodine, 1 mg; zinc, 100 mg; selenium, 0.1 mg.

** Calculated according to NRC.^29^

### Investigated measurements

Throughout the experimental periods, records were kept of the initial body weight, live body weights (LBW), feed intake (FI), and body weight gain (BWG). Rabbits were individually weighed before the morning diet. Further, the feed conversion ratio (FCR) was computed. The performance index of rabbits (PI) was computed as follows: (Final LBW kg/FCR) * 100 = PI.^31^^,^^32^ Following a 12-hour fast, eight rabbits (one from each replicate) were randomly selected for slaughtering, weighed, and then slaughtered, skinned, and eviscerated to evaluate carcass and meat characteristics as soon as the experiment period ended. The Blasco et al.^33^ procedure of the slaughter process and carcass quality was followed. The carcass weight, which was determined by removing the head and visceral organs, was then converted to a proportion of the LBW to determine the dressing ratio. The two front legs (including the thoracic insertion muscles), the loin (including the abdominal wall and the ribs after the seventh thoracic rib), and the hind legs (including the sacral bone and the lumbar vertebra after the sixth lumbar vertebra) were the three cuts made from the carcass.

### Serum biochemical analysis

In clean, dry, sterile tubes, blood samples were taken from rabbits during slaughtering processes and left to clot. Serum samples were collected as explained by Sitohy et al.^34^ then kept at −20 °C till blood analysis procedures. The serum levels of low-density lipoprotein (LDL), high-density lipoprotein (HDL), total cholesterol (TC), triglycerides (TG), alanine aminotransferase (ALT), aspartate aminotransferase (AST) urea, creatinine, total protein (Tp) and albumin (Alb) were valued by employing commercial diagnostic kits provided by Bio Diagnostic Co. (Giza, Egypt), and determined in accordance with the manufacturer’s commands.^16^^,^^35^^,^^36^ Globulin was calculated by the following equations (^37^^,^^38^ 2020). In addition, while testing creatinine and immunological response (IgG, IgM), follow the manufacturer’s recommendations when using commercial kits. The indicators of antioxidant status, e.g., malondialdehyde (MDA), Glutathione peroxidase (GPx), glutathione-S-transferase (GSTs), and Glutathione reduced (GSH) were ascertained using Diamond Diagnostic Co. kits (Giza, Egypt) in accordance with the manufacturer’s instructions.

### Cecal and diet microbial count

At six weeks, the diet samples underwent microbiological testing. In a screw-top bottle, they were mixed with a sterile saline peptone solution at a 1:10 (w/v) ratio, and they were then homogenized for 3 minutes. For every kind of bacterium, a different medium was used. Over two days at 30 °C, the total bacterial count (TBC) was estimated using Plate Count Agar. For about 5 days at a temperature of 25 °C, the Rose Bengal Chloramphenicol Agar was used to determine the total yeast and mold count (TYMC). Using the procedure outlined by Harrigen and McCance-Margaret,^39^ the total number of coliforms was computed on MacConkey agar medium. According to Szabo et al.^40^ *Escherichia coli* (*E. coli*) was counted on Tryptone Bile Glucuronide Agar at 37 °C for 24 hours.

Moreover, the bacterial count in the rabbits’ cecum samples was evaluated as in the rabbits’ diet samples. Rabbits’ cecal samples (five per replicate) were mixed in a screw bottle with sterile saline peptone solutions (1:10 w/v). We prepared decimal serially diluted solutions up to 10^7^. The different bacterial species were counted on specific agar media.^41^ TBC was estimated as mentioned by Sheiha et al.^42^ on Plate Count Agar (PCA). On MacConkey agar medium, total coliforms were counted. *E. coli* was counted at Tryptone Bile Glucuronide Agar at 37 °C for 24 hours as described by Richard et al.^43^ *Salmonella* spp. was estimated at S.S. agar as per Edwards and Hilderbrand^44^; *Salmonella* spp. could be noticed easily through black colonies. Molds were also estimated as described by Ros and Harrison (2012). Lactic acid bacteria were identified using MRS medium and MRS-cysteine agar to increase the isolation of *lactobacilli*, *Bacillus*, and *bifidobacteria*, respectively, according to Argyri et al.^45^

### Meat quality

Three rabbits’ breast samples were gathered and cut into 3-cm pieces. A Hunter Lab spectrophotometer (Vista, Reston, VA, USA) was used to measure the meat samples’ color parameters, specifically lightness (L), redness (a), and yellowness (b), following the procedure outlined by AMSA.^46^ A HANNA pH meter (Woonsocket, USA) was used to measure the breast samples’ pH values at 1, 5, and 10 days of storage.

### Cooking loss

The total weight of the meat samples for each treatment was used to determine their cooking yields. Yields were computed as a proportion of cooked weight divided by raw weight. Cooking loss was evaluated as the weight difference between the individual raw meat and the cooked meat. The values of moisture retention characterize the amount of moisture retained in the cooked product per 100 g of raw sample. It was calculated as described by Kumar and Sharma.^47^

### Water holding capacity (WHC)

The method described by Huff-Lonergan and Lonergan^48^ was used to calculate the WHC; a clean and dry centrifuge tube (50 ml in volume) that had been pre-weighted for each sample contained 5 grams of rabbits’ meat samples, which was a minced mixture of breast and thigh muscles. After that, distilled water was gradually added to the tube while being constantly stirred with a glass rod. The mixture was wetted, then at a speed of 4000 rpm for ten minutes, the mixture was centrifuged. Following centrifugation, the supernatant was removed using decantation. The next equation was employed to compute WHC from the:

WHC=(W2‐W1)/W0

Where: W0 = the sample weight (g); W1 the tube weight + the sample (g); W2 = the tube weight + the sediment (g).

### Statistical analysis

The Shapiro-Wilk test^49^ was employed in order to verify the assumption of data normality. The General Linear Model (GLM) technique was used to examine the data procedure of SPSS,^50^ as shown in the next model:

Yij = μ + Ti + eij,

Where Yij = the individual observation, μ = the overall mean, Ti = the effect of treatments, and eij = the experimental error. Significant differences between treatment means were tested according to Duncan’s multiple test.^51^

## Results

### Rabbit growth performance traits

Throughout the experiment period, LBW, BWG, FI, and FCR parameters were estimated. The impact of probiotics at several levels on these characteristics is summarized in Table 2. The obtained results point to the fact that only FI (7-9 weeks), average FI, average FCR, and PI were meaningfully influenced (P ≤ 0.05) by the tested additives through the experimental period. When compared to the T0 group, it is obvious that T2 achieved the maximum final LBW (9-11 weeks), average BWG, and PI value, along with the best FCR values. Conversely, T1 recorded the best average FI values.

**Table 2.** LBW, BWG, FCR, and PI of New Zealand white rabbits in response to the addition of dietary probiotics.

Items	Treatments			SEM	*P*-value
	T0	T1	T2		
Live body weight					
Initial LBW	844.96	845.02	845.04	1.44	1.000
LBW (5–7 wks)	1231.00	1179.00	1222.00	24.16	0.704
LBW (7–9 wks)	1868.67	1787.00	1838.33	27.08	0.524
LBW (9–11 wks)	2206.00	2154.00	2234.00	18.09	0.195
Body weight gain					
BWG (5–7 wks)	25.73	22.27	25.13	1.61	0.705
BWG (7–9 wks)	39.86	38.00	38.52	0.68	0.582
BWG (9–11 wks)	22.49	24.47	26.38	1.64	0.689
Average BWG	29.59	28.46	30.20	0.37	0.150
Feed intake					
FI (5–7 wks)	82.08	80.83	77.33	1.58	0.507
FI (7–9 wks)	125.56^a^	100.84^b^	118.58^a^	4.47	0.034
FI (9–11 wks)	130.50	120.07	123.60	3.30	0.484
Average FI	112.72^a^	100.58^b^	106.50^ab^	2.21	0.052
Feed conversion ratio					
FCR (5–7 wks)	3.34	3.77	3.08	0.23	0.526
FCR (7–9 wks)	3.17	2.65	3.08	0.12	0.151
FCR (9–11 wks)	6.48	4.93	4.71	0.54	0.400
Average FCR	3.81^a^	3.53^b^	3.53^b^	0.05	0.011
Performance index (PI)	57.91^b^	61.01^ab^	63.34^a^	0.92	0.019

LBW: live body weight; BWG: body weight gain; FCR: feed conversion ratio; PI: performance index.

^a,b,c^ Means with different letters in the row represent significant differences at *P* ≤ 0.05, SEM: Standard Mean Error.

### Slaughtering traits

In Table 3, the impacts of different probiotics doses on the (absolute and relative) carcass weights of New Zealand White rabbits have been displayed. The inclusion of probiotics had a great positive influence on most carcass characteristics. The carcass weight %, forequarter weight %, hindquarter weight (both absolute and %), and kidney weight (both absolute and %) were meaningfully (P ≤ 0.05) affected by the probiotic’s levels. The highest carcass, liver, and dressing weights were obtained from rabbits treated with T2, in comparison to the T1 and T0 groups. Whereas absolute and relative weights of forequarter, hindquarter, and giblets were detected in rabbits of the T1group.These findings propose that incorporating probiotics into the New Zealand White rabbits’ diet not only boosts their overall LBW but may also enhance carcass characteristics.

**Table 3.** New Zealand White rabbits’ carcass weights (g) in response to the addition of dietary probiotics.

Items	Treatments			SEM	*P*-value
	T0	T1	T2		
Carcass	1077.00	1077.00	1106.00	8.33	0.292
Carcass%	50.68^b^	52.54^a^	50.99^b^	0.35	0.037
Forequarter	327.00	393.00	366.00	12.95	0.093
Forequarter %	15.39^b^	19.16^a^	16.86^ab^	0.65	0.024
Loin	317.00	275.00	297.00	8.04	0.080
Loin%	14.92	13.41	13.70	0.34	0.164
Hindquarter	419.00^b^	468.00^a^	433.00^b^	7.72	0.001
Hindquarter %	19.72^b^	22.83^a^	19.97^b^	0.51	0.000
liver	72.00	72.50	75.00	2.09	0.859
liver%	3.39	3.54	3.46	0.11	0.879
Heart	7.00	9.00	8.00	0.47	0.244
Heart%	0.33	0.44	0.37	0.02	0.145
Kidney	19.00^a^	18.50^a^	17.00^b^	0.35	0.021
Kidney%	0.89^a^	0.90^a^	0.78^b^	0.02	0.003
Giblets	98.00	100.00	100.00	2.36	0.941
Giblets%	4.61	4.88	4.61	0.12	0.660
Dressing	1175.00	1177.00	1206.00	9.34	0.361
Dressing%	55.30	57.41	55.60	0.45	0.091

^a,b,c^ Means with different letters in the row represent significant differences at *P* ≤ 0.05, SEM: Standard Mean Error.

### Blood biochemical parameters

Blood indices, including liver and kidney biomarkers, protein fraction, lipid profile, immunoglobulins, as well as antioxidant enzymes, mirror the nutritional and health status of animals. As exhibited in Table 4, probiotic inclusion in NZW rabbit diets increased liver function enzymes; specifically, groups T1 and T2, which gained the highest levels of ALT and AST, respectively, compared to the T0 group. Kidney functions were meaningfully (P ≤ 0.05) diminished due to probiotic treatments. The lowest levels of urea and creatinine were detected in the rabbit group of T2. The same level of probiotic treatment (T2) exhibited the highest level of total protein, accompanied by a reduction in albumin and globulin levels compared to the T1 and T0 groups. Furthermore, the increase in probiotics addition levels enhanced the NZW rabbit Lipid profile, as evidenced by a reduction in TC, TG, and LDL levels, accompanied by an increase in HDL levels. Again, T2 acquired the lowest levels of TC, TG, and LDL, and maximum levels of HDL. Additionally, antioxidant status was significantly improved (P ≤ 0.05) due to probiotic treatments; the T2 treatment led to a minimum level of MDA, with a maximum level of GPx, GSTs, and GSH compared to T1 and T0 groups. The immune system produces proteins called immunoglobulins, or antibodies, to recognize and destroy foreign substances like bacteria and viruses. Probiotic addition to rabbits’ diets improves their immune system, which was proven in our study by an increase in IgG and IgM, with increased probiotic levels in comparison to T0.

**Table 4.** Blood indices of New Zealand white rabbits as in response to the addition of dietary probiotics.

Items	Treatments			SEM	*P*-value
	T0	T1	T2		
Liver function					
ALT(IU/L)	13.26	13.27	13.36	0.10	0.910
AST(IU/L)	112.32	113.05	112.67	0.37	0.776
Kidney function					
Urea(mg/dl)	39.99^a^	35.35^ab^	32.43^b^	1.33	0.030
Creatinine(mg/dl)	0.59^a^	0.48^b^	0.44^c^	0.02	0.001
Protein fraction					
Total protein (mg/dl)	6.64^a^	6.52^b^	6.49^b^	0.03	0.040
Albumin (mg/dl)	3.23	3.11	3.11	0.03	0.130
Globulin (mg/dl)	3.41	3.41	3.37	0.04	0.915
A/G ratio	0.95	0.91	0.92	0.02	0.682
Lipid profile					
TC (mg/dl)	61.53	60.16	58.53	0.66	0.180
TG (mg/dl)	89.66	88.33	87.24	0.57	0.243
HDL (mg/dl)	28.96^c^	30.38^b^	32.42^a^	0.54	0.002
LDL (mg/dl)	14.29^a^	12.11^ab^	8.66^b^	0.96	0.021
Antioxidant enzymes					
GPx (nmol /mL)	166.67	161.67	168.67	1.94	0.363
GSTs (nmol /mL)	4.88^b^	5.88^a^	5.53^a^	0.17	0.016
GSH (nmol /mL)	74.56^b^	75.67^b^	78.48^a^	0.68	0.018
MDA (nmol/mL)	3.92^a^	3.66^b^	3.36^c^	0.08	0.000
Immunoglobulins					
IgG (mg/mL)	212.75	222.33	226.97	4.03	0.388
IgM(mg/mL)	16.50	16.91	17.32	0.16	0.075

A/G ratio: albumin to globulin ratio; TC: total cholesterol; TG: triglycerides; HDL: high-density lipoprotein; LDL: low-density lipoprotein; GPx: Glutathione peroxidase; GSTs: glutathione-S-transferase; GSH: Glutathione reduced; MDA: malondialdehyde; IgG: immunoglobulin G; IgM: immunoglobulin M.

^a,b,c^ Means with different letters in the row represent significant differences at *P* ≤ 0.05, SEM: Standard Mean Error.

### Meat quality

Table 5 illustrates how the meat quality of NZW rabbits responds to probiotics addition at different levels. Substantial alterations (P ≤ 0.05) between all treatments in meat pH values on the 5^th^ and 10^th^ day were observed. The lowest pH values of rabbit meat were acquired by the T2 group, in comparison to other treatments. Further, WHC decreased significantly (P ≤ 0.05) whenever the probiotics level increased; in comparison to the T0 group, the group T1, followed by T2, exhibited the lowest WHC values. In addition, the lowest values of purge and cooking loss were obtained from the T2 and T1 groups, respectively. Probiotics treatments improved the color measurement values of NZW rabbit meat. The T1 group presented the maximum values of lightness (L*) and the lowest value of redness (a*), while the minimum values of yellowness (b*) were found in the T2 group in comparison to the T0 group.

**Table 5.** Meat quality of New Zealand white rabbits in response to the addition of dietary probiotics

Items	Treatments			SEM	*P*-value
	T0	T1	T2		
pH (1 day)	6.08	6.13	5.98	0.05	0.466
pH(5^th^day)	6.35^a^	6.39^a^	6.22^b^	0.03	0.001
pH(10^th^day)	6.62^a^	6.69^a^	6.46^b^	0.04	0.025
WHC	60.79^a^	43.74^b^	54.37^ab^	3.12	0.048
Purge	4.45	4.01	3.17	0.43	0.527
Cooking loss	24.22	22.73	23.44	0.64	0.696
Color parameters					
Lightness (L*)	55.03	59.98	51.42	2.88	0.542
Redness (a*)	25.28	20.21	24.06	2.77	0.788
Yellowness (b*)	31.15	26.31	24.20	1.61	0.209

WHC: water holding capacity.

^a,b,c^ Means with different letters in the row represent significant differences at *P* ≤ 0.05, SEM: Standard Mean Error.

### Cecal and dietary bacterial load

The impact of different probiotic levels on the cecal and diet microbial load of NZW growing rabbits was summarized in Table 6. There were substantial (P ≤ 0.05) differences detected among all treatments for all cecal bacterial populations, except for the *Bacillus* count. Probiotic addition has improved the cecal microbiota of NZW growing rabbits. In comparison to the T2 and T0 groups, T1 showed the lowest values of TBC and pathogenic bacteria (*Salmonella,* Coliform*, and E. coli*). In contrast, T2 exhibited the highest count of beneficial bacteria (including *Lactobacillus* and *Bacillus*), while the T0 group acquired the lowest total yeast count (TYC). Regarding the diets’ bacterial load, the highest *Lactobacillus* and *Bacillus* count was observed in the T0 group, followed by the T1 Group, respectively. Conversely, the lowest TBC and TYC count was found in the T1 rabbits’ group.

**Table 6.** Cecal and dietary bacterial load of New Zealand white rabbits in response to the addition of dietary probiotics

Items	Treatments			SEM	*P*-value
	T0	T1	T2		
Cecal bacterial load					
TBC	8.92^a^	8.51^b^	8.51^b^	0.08	0.008
Lactobacillus	6.27^b^	6.77^a^	6.85^a^	0.09	0.001
Bacillus	5.66	5.61	5.73	0.06	0.753
Salmonella	4.90^a^	4.38^b^	4.40^b^	0.09	0.000
Coliform	7.19^a^	6.60^b^	6.66^b^	0.08	0.003
E. coli	6.82^a^	5.44^b^	5.56^b^	0.24	0.002
TYC	2.25^c^	2.51^b^	3.62^a^	0.21	0.000
Dietary bacterial load					
TBC	3.70^a^	2.11^c^	2.36^b^	0.15	<0.001
Lactobacillus	2.61^a^	1.75^b^	1.68^b^	0.12	<0.001
Bacillus	2.26^b^	2.39^a^	2.42^a^	0.23	0.006
TYC	1.60^a^	1.47^c^	1.54^b^	0.16	<0.001

TBC: total bacterial count; TYC: total yeast count.

^a,b,c^ Means with different letters in the row represent significant differences at *P* ≤ 0.05, SEM: Standard Mean Error.

## Discussion

The effects of probiotics on rabbit husbandry have been the focus of numerous published studies in recent decades. Since the study articles cover a wide range of topics, including rabbit species, sex, ages, types of probiotics, types of basal meals, trial duration, ambient circumstances, etc., in our study, live body weight, body weight gain, FI, FCR, and PI of NZW growing rabbits were improved with probiotic treatments. In treated rabbits, *Lactobacillus acidophilus* or *Lactobacillus lactis* improved the FCR value and average daily BWG.^52^ Further, our findings align with El-Sawy et al.,^22^ who noted that adding probiotics to rabbit drinking water boosted productivity and feed utilization while having no detrimental impact on carcass characteristics. This may be related to the fact that the probiotic addition to rabbits reduces disease incidence and reinforces the immune system of treated rabbits.^19^ Three distinct probiotic species (*L.rhamnosus*, *B. animalis* subsp. *Lactis,* and *S. boulardii*) were studied in rabbits by ^53^^,^^54^ (2021); regardless of the particular strain of probiotics used in the diet, the results demonstrated a significant increase in average daily BWG levels.

Additionally, giving probiotics may boost the cecum’s population of some beneficial bacteria that can generate butyrate. By supporting nutrition and preserving the structural integrity of intestinal villi, butyric acid regulates the activities of the large intestine. Additionally, it affects mucin structure and goblet cell differentiation, which improves the function of the mucous barrier and digestive processes.^55^^,^^56^ It is believed that the digestive enzymes production e.g., lipase, protease, and amylase, which in turn enhances food digestibility, is what causes the improvement in productive metrics of rabbits given probiotics.^52^^,^^57^ Moreover, weaned rabbits fed *Clostridium butyricum* showed improved weight gain due to its probiotic action on small intestine architecture and digestive enzymes. In the rabbits’ digestive tract, the probiotic complex can increase and synthesize a varied range of digestive enzymes. It employs different carbohydrates to produce volatile fatty acids (VFAs), in that way extending the rabbit’s fermentative gastrointestinal system and playing a role in maintaining the intestinal tract healthy.^58^^,^^59^

A previous investigation by Xia et al.^21^ on Hyla rabbits suggested that diet supplementation with probiotics (heat-killed *Lactobacillus acidophilus*) at doses of 0.6, and 0.8 g/kg improved rabbits’ feed efficiency and growth performance traits; the observed boost in the protease, lipase, and cellulase activity in the rabbits’ intestinal tract is consistent with the enhancement in their feed efficiency.^60^ In research by Ashour et al.^18^ noted that in New Zealand White (NZW) rabbits that received probiotics (1 ml of *Lactobacillus acidophilus* and 1 ml of *Bifidobacterium bifidum*)/kg diet had significantly acquired higher PI than those of the control group, which is consistent with our findings. In comparison to the untreated group, Italian White growing rabbits receiving a diet provided with 250 mg of probiotic mixture/kg showed enhanced feed efficiency as well as a noticeably greater average daily BWG and final body weight.^24^ Noted improvement in performance traits may be related to probiotic inclusion in diets, which enhances nutrient availability and absorption in the gut, as well as leading to reduced feed consumption.^61^

On the contrary, when a particular probiotic (Saccharomyces cerevisiae) was given to NZW rabbits in a study by Emmanuel et al. (2025) at a level of 0.120 g/kg of LBW, the rabbits displayed no changes in their LBW, DWG, or FCR values. Also, Simonová et al.^62^ and Li et al.^63^ indicate insignificant changes in growth performance traits of rabbits treated with different levels of probiotics.

As mentioned in the recent study, probiotics had significantly improved most of the absolute and relative weights of NZW rabbits’ carcasses. Notably, our obtained data are close to those obtained by Mohamed et al.^64^ who found that probiotics-supplemented diets, specifically *Bacillus subtilis* and a combination of *L. acidophilus* and *B. bifidum*, improve the majority of the studied rabbits’ carcass characteristics. In addition, Fathi et al.^65^ exhibited that rabbits fed diets with *B. subtilis* at levels 0.2 and 0.4 mg/t enhanced the carcass weight (only the level of 0.4 mg/t), dressing percentage, and carcass cuts of the middle and hind parts as a proportion of LBW.

As well as Tufarelli et al.^24^ noted that slaughter weight as well as carcass dressing yield were enhanced when Italian White growing rabbits received 250 mg of probiotic mixture/kg of diet. Too, a previous study by Mohamed et al.^64^ noted that the inclusion of varying levels of *B. bifidum*, *L. acidophilus*, a combination of bacteria, and *S. cerevisiae* in NZW and Baladi Black (BB) rabbits’ diets enhanced most of their carcass characteristics; BB rabbits fed *Bifidobacterium bifidum* supplements resulted in higher carcass weight, carcass yield %, giblets and heavier internal organs weights. Probiotics can increase rabbit meat production, especially in prime cuts such as those in the middle and back. That may be linked to probiotics, which may aid in nutrition absorption by promoting gut health.^66^

In contrast, Hegab et al.^67^ and El-Sawy et al.^22^ didn’t note any impacts of dietary probiotics on the carcass traits among different treatments. Many prior studies demonstrated that there were no impacts on carcass characteristics owing to diverse treatments with various levels of probiotics, such as the studies published by Abd El-Moneim et al.^68^, De-Souza et al.^69^, and Abou-Kassem et al.^41^

Because it makes it easier for various metabolites to move throughout the body, blood is vital to the internal environment. To a certain degree, biochemical markers can shed light on the body’s metabolic processes. In the existing study, probiotic-supplemented diets in general significantly (P < 0.05) altered serum biomarkers of kidney function. Our findings were in line with Alqahtani et al.,^70^ who noted that levels of creatinine and uric acid were diminished due to *Bacillus toyonensis* supplementation with different doses in the broilers’ diet. In terms of liver function, according to Abd^71^ broilers given probiotics at a level of 10.0 ml/liter of drinking water had considerably higher levels of liver enzymes (ALT and AST) than those in the untreated group. Conversely, Zhao et al.^72^ noted that female rabbits supplemented with 3, 6, and 9 g probiotic mixture/kg diet from day 24 of gestation till weaning day did not significantly alter plasma AST and ALT levels.

Whereas Abdelkader et al.^73^ noted that broiler-fed diets supplemented with probiotics recorded significantly lower ALT and AST levels than those of the control group. Also, our study detected a reduction in Tp, globulin, and Alb levels due to probiotic additions. These results differ from the results obtained by Kurchaeva et al.^74^ who found a rise in levels of Tp, Alb, and globulin when rabbits were fed diets provided with 1 and 0.6 g probiotic preparation “Sporotermin” per kg diet.

El-Shafei et al.^75^ reported that when rabbits’ diets were supplemented with probiotics (*L. plantarum*), there was a noteworthy reduction in serum levels of TG and TC. Probiotics appear to have a hypocholesterolemic impact through a variety of mechanisms. These bioactive compounds can bind or incorporate intestinal cholesterol into bacterial cells, which then use it to create short fatty acids (SFA) for cellular metabolism.^76^ Also, according to a study by Kadja et al.^77^ three rabbit groups fed with 3 probiotic strains (*Lactobacillus rhamnosus*, *Bifidobacterium animalis* subsp. *lactis*, and *S. boulardii*) showed a substantial decrease in TC and TG levels. The lipid profile of weaned rabbits had improved when their diets were provided with a probiotic (*Bacillus* spp. mixture) as reported by Khalifa et al.^58^

Compared to the untreated group, rabbits that were fed a diet provided with 250 mg of probiotic mixture/kg had significantly decreased (p < 0.005) serum levels of LDL, HDL, TC, and TG.^24^ Improving lipid profile may be related to these microbial strains that can block the hydroxyl methylglutaryl CoA enzyme, which plays a role in cholesterogenesis, and convert cholesterol into coprostanol.^78^^,^^79^) Probiotics also reduce the production of cholesterol through reducing the activity of (the HMG-CoA enzyme), which is the enzyme linked to cholesterol creation; Furthermore, probiotics may inhibit the intestinal recycling of bile salts; as a result, an additional cholesterol is needed to produce bile acids, which reduces the level of cholesterol in the blood, as pointed out by Alqahtani et al.^70^

Superoxide dismutase (SOD), Glutathione peroxidase (GSH-PX), catalase (CAT), and other enzymes make up the enzymatic antioxidant system of the body. It is commonly known that two essential endogenous antioxidant enzymes, CAT and SOD, are essential in halting oxidative damage. A cellular damage measurement, particularly lipid damage brought on by oxidative stress from free radicals, is MDA, a byproduct of cellular membrane peroxidation. Research has shown that *Bifidobacterium* and *Lactobacillus* can both *in vivo* and *in vitro* exhibit antioxidant features and diminish oxidative damage in rabbits.^80^ A high level of Clostridium butyricum in weaning rabbits led to a reduction in the amount of malondialdehyde (MDA) in intestinal tissues and a rise in CAT, GSH-Px activities, and SOD. This implies that consuming probiotics boosted intestinal capacity for antioxidants, which in turn led to better gut health.^59^

In rabbits with diet-ochratoxin A-induced toxicity, *Aspergillus awamori* treatment led to elevated SOD and CAT enzyme activity, which efficiently eliminated excess reactive oxygen species (ROS) and improved the treated group’s antioxidant resistance.^81^ Further, Xia et al.^21^ found that in Hyla rabbits suggested that diet supplementation with probiotics (heat-killed *Lactobacillus acidophilus*) at levels of 0, 0.2, 0.4, 0.6, and 0.8 g/kg, the levels of 0.6 and 0.8 g/kg revealed a significant (p < 0.05) rise in the serum SOD activity, however, serum T-AOC activity was suggestively (p < 0.05) raised in the addition level of 0.8 g/kg. On the other hand, both GSH-Px and MDA levels in serum are not affected by probiotics treatment. Too, Tufarelli et al.^24^ found considerably (p < 0.005) increased CAT, GST, GPx, and SOD levels, compared to the untreated group, when Italian White growing rabbits were provided with 250 mg of probiotic mixture/kg diet. Short-chain fatty acids produced by probiotics play a role as Keap1-Nrf2 (Nrf2) activators. Nrf2 is positively associated with T-AOC, SOD, and GSH-Px production.^82^^,^^83^

Immunoglobulins, such as IgG, IgA, and IgM, are essential components of the immune response in the body, and their levels are positively connected with an individual’s immunocompetence. Findings in the existing study proved that probiotics increased IgG and IgM, which is confirmed in an investigation by Wang et al.^84^ who claimed that IgM and IgG levels were markedly raised when rabbits were fed diets provided with probiotics (*Lactobacillus zeae* and *Lactobacillus casei*). Daily probiotic supplementation (*E. faecium* R1 and *Ligilactobacillus animalis* R2) markedly (p < 0.05) increased serum content of IgA, IgG, and IgM levels in New Zealand rabbits recently weaned.^63^ Previous research by Xia et al.^21^ on Hyla rabbits suggested that diet supplementation with probiotics (heat-killed *Lactobacillus acidophilus*) at doses of 0, 0.2, 0.4, 0.6, and 0.8 g/kg meaningfully increased (p < 0.05) IgA and IgM levels in comparison to the untreated group. In addition, probiotic mixture addition at levels of 3, 6, and 9 g/kg diet for rabbits significantly boosts the IgG, IgA, and IgM levels compared to untreated groups.^72^ Moreover, by encouraging the creation of ATP and fatty acid content in B cells, short-chain fatty acids (produced by probiotics) can speed up the stimulation, differentiation, and antibody generation by B cells; this will increase the release of IgA and IgM.^85^^,^^86^

Additionally, laying hens that got 30% single-cell protein (SCP) replacement in combination with 50 or 100 mg/kg probiotic showed the highest villus height to crypt depth ratio, antibody titer against sheep red blood cells (in secondary response), and Newcastle disease (ND) vaccine.^87^ Furthermore, bacteria in the colon break down carbohydrates and proteins, producing a significant amount of short-chain fatty acids, which in the cecum can lower the intestinal tract pH level, inhibit dangerous bacteria growth, advance the function of the intestinal epithelial barrier, influence immunity, and reduce inflammation.^88^

Rabbit meat quality varies depending on the fattening period, rabbit breed, and slaughter methods. Regarding the influence of probiotics on meat quality traits, our research findings indicates that tested probiotic had improved meat quality traits of NZW rabbits, a similar outcomes obtained in quails by Abou-Kassem et al.^41^ who confirmed that growing quail fed diets supplemented with different levels of probiotics (*Bacillus toyonensis* and *Bifidobacterium bifidum*) reduced cooking loss %, proteolysis (%), and increased WHC%, besides, values of meat pH were augmented with probiotic addition at levels 0.75,1.0 ml *B. toyonensis* and level of 1.0 ml *B. bifidum*/kg of quail diets compared to untreated birds. A previous study by Saleh et al.^89^ noted that adding bacteriophage at a level of 1 kg/t to broilers’ diet enhanced the meat’s L*, b*, and oxidation. Also, the Lactobacillus and lactic acid bacteria count was considerably raised by the addition of bacteriophage compared to the control group.

On the other hand, Tufarelli et al.^24^ found insignificant impacts of probiotic treatments on values of Italian White rabbits’ meat quality when their diets were provided with 250 mg of probiotic mixture/kg. Moreover, Pogány Simonová et al.^90^ demonstrated no changes in meat quality traits of Hyplus breed rabbits, including pH (after 48 h), color values, proximate composition, or WHC%, when rabbits were provided with a dosage of 500 µL/rabbit/day probiotic (*Enterococcus faecium*) in drinking water. Likewise, Rotolo et al.^91^ found no discernible impact of live Saccharomyces cerevisiae boulardii treatment on the proximate composition, meat color, pH, and cooking loss % of longissimus dorsi muscle.

The principal and most complicated microecological system found in animals is the intestinal microbiota, which colonizes the body from the time of birth and remains there throughout life. It is important to remember that this probiotic mixture might be able to inhibit potentially harmful germs like *Coliform* and *Clostridium*, because the probiotic mixture’s inhibitory modes of action differ from those of single-strain probiotics, and it seems to be more effective at preventing intestinal infections.^92^ The consequent alteration of the gut’s physico-chemical environment might prevent the implantation of harmful bacteria by dropping the pH or O_2_ levels in the gastrointestinal system. a decrease in intestinal *coliform* count, a rise in population of cecal *lactobacilli*, and an upsurge in cecal acetic acid levels in rabbits treated with *Lactobacillus acidophilus*, either lonely or in mixture with *Bacillus subtilis* was reported in a previous study by Phuoc and Jamikorn.^57^

Additionally, Zhu et al.^93^ detected that the addition of inactivated probiotics at a dose of 0.5 g/kg to the broiler diet positively improved their cecum microbiota. Prior studies showed that cecal *lactobacilli* were more common in rabbits fed diets enriched with *Lactobacillus acidophilus*, indicating that, more than other bacteria, *Lactobacillus* species have a detrimental impact on the ability of pathogenic bacteria to invade and proliferate.^56^^,^^67^ Because multi-strain bacterial colonization can strengthen the microbiota community’s tolerance to environmental stresses and infections, the high degree of diversity it fosters may be helpful.^56^

In addition, Helal et al.^76^ discovered an upsurge in *Lactobacillus* spp. count accompanied by a reduction in *Clostridium perfringens* and *Escherichia coli* in rabbits that fed a probiotic mixture with *Saccharomyces cerevisiae* and *B. subtilis*. The intestinal bacterial diversity was found to be improved by probiotic treatment, supporting a higher absolute richness of beneficial species of bacteria (e.g., *Bacillus* and *Lactobacillus)* in the intestinal tract.^94^^,^^95^ Additionally, Khalifa et al.^58^ and Wlazło et al.^96^ observed an increase in *lactobacilli* as a result of probiotic addition to rabbits’ diet, and they discovered that the rabbits’ digestive tract had more lactic acid bacteria with fewer *coliforms*. In Italian White growing rabbits fed diets containing 250 mg of probiotic mixture/kg, resulted in a substantial decline in counts of cecal *E. coli*, *Bacillus* spp., and *Clostridium* spp., whereas *Lactobacillus* spp. and *Bacterioides* spp. counts were significantly increased; moreover, the probiotic mixture supplemented groups had higher butyric acid and acetic acid concentrations in the rabbits’ cecum than those of the control group. Organic acids produced by microbiota during glucose fermentation suggest enhanced microbial growth and cecum fermentation activity.^24^

## Conclusion

In summary, supplementing the diets of New Zealand White rabbits with a combination of *Bacillus velezensis* and *Lactococcus lactis* significantly improved their growth performance, carcass traits, and overall physiological health. The most pronounced benefits were observed at the higher supplementation level (2 ml/kg of each probiotic strain), which led to better feed efficiency, enhanced blood biochemical profiles, and a healthier microbial balance in the gut. The probiotic-treated groups also showed stronger immune responses and greater antioxidant capacity, indicating improved resilience and well-being. Notably, the quality of the meat was enhanced, with better color characteristics, reduced water loss during cooking, and improved water-holding capacity—traits that contribute to more desirable and marketable meat products. The reduction of harmful gut bacteria alongside an increase in beneficial microbial populations underscores the role of probiotics in supporting digestive health. These results suggest that probiotic supplementation is a safe and effective alternative to antibiotics, offering a sustainable and health-oriented strategy for rabbit production that aligns with consumer demands for high-quality, antibiotic-free meat.

## Data Availability

Data will be available upon reasonable request from the corresponding authors.
